# Characterization of murine experimental autoimmune encephalomyelitis induced by active immunization with a CD8 epitope of myelin oligodendrocyte glycoprotein

**DOI:** 10.1186/1479-5876-10-S3-P26

**Published:** 2012-11-28

**Authors:** Flora Guillot, Sophie Brouard, David A Laplaud, Arnaud B Nicot

**Affiliations:** 1 Institut National de la Santé et de la Recherche Médicale UMRS 1064 / ITUN, Nantes, France; 2 L'UNAM, University of Nantes, Nantes, France

## 

Multiple sclerosis (MS) is an autoimmune, demyelinating and degenerative disease of the central nervous system (CNS). Anti-myelin CD4 T cells are strongly associated with disease development in several animal models of MS such as experimental autoimmune encephalomyelitis (EAE). However, CD8 T cells often outnumber CD4 T cells in the CNS parenchyma of MS patients and recent studies suggest that anti-myelin CD8 T cells may be also implicated. In order to better understand the contribution of pathogenic CD8 T cells, C57Bl/6 female mice were immunized with an epitope of myelin oligodendrocyte protein (MOG) specifically presented by MHC-I to CD8 T cells, as reported by Ford & Evavold in 2005. Only one third of these mice developed EAE with mild clinical signs. In contrast, mice immunized with MOG_35-55_ developed hindlimb paralysis, as expected for this model.

Proliferation and FACS analysis of T cell reactivity using splenocytes isolated from CD8 epitope-immunized mice confirmed the emergence of specific MOG-reactive CD8 T cells *in vivo*.

Immunohistochemical analysis of the spinal cord, cerebellum and optic nerve in mice that developed the first clinical signs indicates that CD8 T cells infiltrate the CNS white matter with a caudo-rostral gradient. Strikingly, the CD8 T cell infiltration is sparse compared with CD4 T cells. Sudan Black staining indicates myelin loss in the CNS white matter of these mice. Further characterization of the CNS T cells in this model is underway. Taken together, these data indicate that anti-myelin CD8 T cells can initiate EAE supporting an early role of CD8 T cells in MS pathogenesis. However, the development or phenotype of autoreactive T cells obtained with this immunization protocol is insufficient to trigger full EAE as compared with the classic EAE model using the CD4 (and CD8) epitope MOG_35-55_.

Supported by Region Pays-de-la-Loire (ABN).

